# Memristor-CMOS Hybrid Neuron Circuit with Nonideal-Effect Correction Related to Parasitic Resistance for Binary-Memristor-Crossbar Neural Networks

**DOI:** 10.3390/mi12070791

**Published:** 2021-07-01

**Authors:** Tien Van Nguyen, Jiyong An, Kyeong-Sik Min

**Affiliations:** School of Electrical Engineering, Kookmin University, Seoul 02707, Korea; tiennv@kookmin.ac.kr (T.V.N.); sunday1903@kookmin.ac.kr (J.A.)

**Keywords:** neuron circuit, nonideal-effect correction, binary memristor crossbar, neural networks, edge intelligence

## Abstract

Voltages and currents in a memristor crossbar can be significantly affected due to nonideal effects such as parasitic source, line, and neuron resistance. These nonideal effects related to the parasitic resistance can cause the degradation of the neural network’s performance realized with the nonideal memristor crossbar. To avoid performance degradation due to the parasitic-resistance-related nonideal effects, adaptive training methods were proposed previously. However, the complicated training algorithm could add a heavy computational burden to the neural network hardware. Especially, the hardware and algorithmic burden can be more serious for edge intelligence applications such as Internet of Things (IoT) sensors. In this paper, a memristor-CMOS hybrid neuron circuit is proposed for compensating the parasitic-resistance-related nonideal effects during not the training phase but the inference one, where the complicated adaptive training is not needed. Moreover, unlike the previous linear correction method performed by the external hardware, the proposed correction circuit can be included in the memristor crossbar to minimize the power and hardware overheads for compensating the nonideal effects. The proposed correction circuit has been verified to be able to restore the degradation of source and output voltages in the nonideal crossbar. For the source voltage, the average percentage error of the uncompensated crossbar is as large as 36.7%. If the correction circuit is used, the percentage error in the source voltage can be reduced from 36.7% to 7.5%. For the output voltage, the average percentage error of the uncompensated crossbar is as large as 65.2%. The correction circuit can improve the percentage error in the output voltage from 65.2% to 8.6%. Almost the percentage error can be reduced to ~1/7 if the correction circuit is used. The nonideal memristor crossbar with the correction circuit has been tested for MNIST and CIFAR-10 datasets in this paper. For MNIST, the uncompensated and compensated crossbars indicate the recognition rate of 90.4% and 95.1%, respectively, compared to 95.5% of the ideal crossbar. For CIFAR-10, the nonideal crossbars without and with the nonideal-effect correction show the rate of 85.3% and 88.1%, respectively, compared to the ideal crossbar achieving the rate as large as 88.9%.

## 1. Introduction

Neural networks draw many interests nowadays, as they have been verified useful in various cognitive tasks such as natural language processing, image recognition, object classification, etc. [1,2,3]. As the applications of neural networks become more complex, the demand for high-performance computing becomes increasingly more. For meeting the need for heavy computation capability, general-purpose digital systems based on CMOS technology have been used widely so far. However, CMOS device scaling has slowed down recently and VDD scaling becomes no longer effective in reducing power consumption [4,5]. Moreover, in terms of computing architecture, the traditional Von Neumann machine has been suffering the memory bottleneck problem [6,7,8,9]. The bottleneck problem becomes more severe especially for computation of neural networks, where memory access for large amounts of data occurs frequently between memory and computing units [10].

One of the important applications of neural networks can be found in edge intelligence such as Internet of Things (IoT) sensors and edge devices, where a massive number of various sensors collect huge amounts of unstructured data everywhere and every time to make human life more comfortable and safe [11,12,13]. If all the data sensed from IoT sensors and edge devices are sent to data centers, amounts of energy for communication and computation for the data centers may be exploding as much as an unbearable level. To avoid the explosion of communication and computing energy at the cloud servers, energy-efficient computing is indispensable in implementing neural networks at IoT sensors and edge devices for edge intelligence [13].

To achieve both high-performance and energy-efficient computing for edge intelligence, memristor crossbars can be considered as good candidates of computing hardware for possible applications such as neural networks, neuromorphic computing, processing-in-memory, etc. [14,15,16]. Memristors demonstrated experimentally in 2008 [17] are nonvolatile memories, where both binary and multi-level values can be stored [18,19,20]. For the architecture, memristor crossbars can be a built-in 3-dimensional multi-layer structure that seems very similar to the biological neuronal structure observed in the human brain [21,22,23,24]. For the fabrication process, Back-end-of-line (BEOL) has been reported in many kinds of literature, to make it possible for memristors fabricated with CMOS devices on the same wafer [10,25,26]). The crossbars made of memristors can perform low-power, parallel, and binary/multi-valued computation similarly with the biological nervous systems. From these properties mentioned above, the crossbars can be considered very suitable particularly for the computational acceleration of neural networks at the edge [20,27].

Vector Matrix Multiplication (VMM) is a core computational function used in both the training and inference phases of neural networks. The VMM operation can be realized in memristor crossbars, where a matrix multiplication operation can be performed using the memristor’s voltage-current relationship according to Ohm’s law [10,28]. The VMM carried out by the memristor crossbar is one example of “computing by physics”. One big advantage of the memristor-crossbar VMM is that the computing capability can be expanded in parallel by adding more columns to the memristor crossbar. The parallel computing of the memristor crossbar is desirable for handling the heavy computational load of VMM operation in data-centric processing systems such as deep-learning neural networks, etc. Moreover, the memory access bottleneck of the Von Neumann architecture can be alleviated significantly in the VMM, because both the memory and computing functions can be merged in the memristor crossbar [10]. In the traditional computing systems, the data fetching and updating operations should take place frequently between the computing and memory units, which are separated in the physical distance as long as ~mm. One more thing to comment here is that memristor’s non-volatility can help IoT sensors extend battery lifetime very long because the synaptic weights of neural networks can be maintained very long time in the crossbar even during the power-off time, without refreshing the stored data.

**Figure 1:**
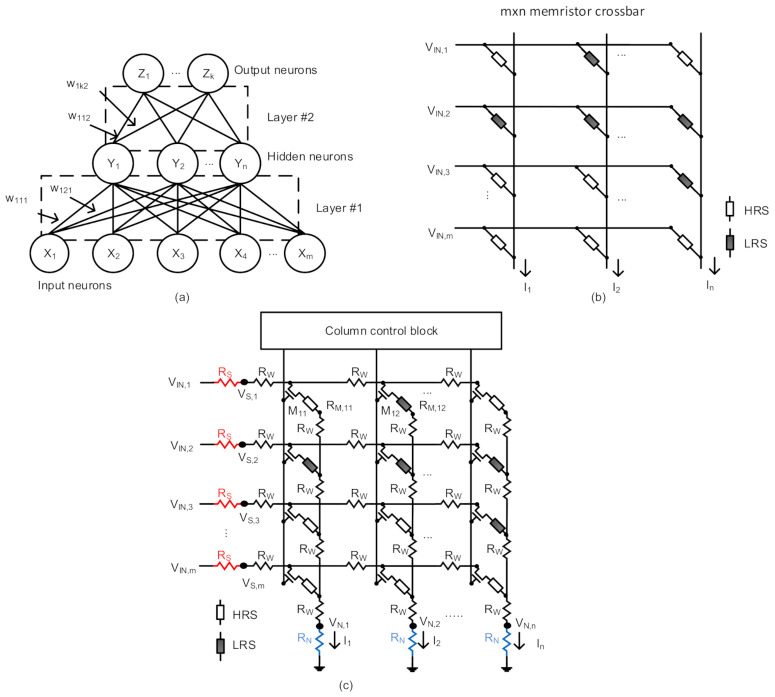
(**a**) Conceptual schematic of two-layer neural network with input, hidden, and output neurons. (**b**) Schematic of ideal memristor crossbar circuit with HRS and LRS memristor synapses. The numbers of rows and columns are ‘m’ and ‘n’, respectively. The parasitic resistance such as source, line, and neuron resistance are assumed zero, in the ideal crossbar. (**c**) Schematic of nonideal memristor crossbar circuit with parasitic crossbar resistance such as source, line, and neuron resistance.

Figure 1a shows a conceptual schematic of Artificial Neural Network (ANN) which is composed of neurons and synaptic connections. Here *X*_1_, *X*_2_, etc. represent input neurons. *Y*_1_, *Y*_2_, etc. are hidden neurons between input and output neurons. *Z*_1_, *Z*_2_, etc. are output neurons of the network in Figure 1a. Here ‘m’, ‘n’, and ‘k’ are the numbers of input, hidden, and output neurons, respectively. The hidden-neuron layers can be more than one. *W*_111_ means a synaptic weight between two neurons, *X*_1_ and *Y*_1_ for the first synapse layer. *W*_121_ is a weight between *X*_1_ and *Y*_2_. Similarly, *W*_112_ is a synapse between *Y*_1_ and *Z*_1_ for the second synapse layer. *W*_1*k*2_ is between *Y*_1_ and *Z_k_*. The hidden neurons, *Y*_1_, *Y*_2_, etc. can be calculated with Vector-Matrix Multiplication (VMM) of the input-neuron vector and the 1st-layer weight matrix. The output neurons, *Z*_1_, *Z*_2_, etc. are obtained from the VMM operation of the hidden-neuron vector and the 2nd-layer weight matrix. The VMM operation can consume a large amount of computing power if the VMM is performed using digital CMOS logic.

Figure 1b shows a schematic of the ideal memristor crossbar with HRS and LRS memristor cells. HRS and LRS mean High Resistance State and Low Resistance State of memristors, respectively. HRS and LRS are represented with white and gray boxes, respectively. Here the memristor crossbar in Figure 1b is used to store the synaptic weights of ANN in Figure 1a. The synaptic weights stored in the crossbar are assumed binary (−1 and +1) or ternary (−1, 0, and +1) in this paper. *V*_*IN*,1_, *V*_*IN*,2_, and *V*_*IN*,*m*_ are input voltages applied to Row #1, Row #2, and Row #m, respectively. *I*_1_, *I*_2_, and *I_N_* are column currents for Column #1, Column #2, and Column #n, respectively. In this figure, ‘m’ and ‘n’ are the numbers of rows and columns in the crossbar, respectively. Parasitic crossbar resistance such as line resistance, neuron resistance, and source resistance are not considered in Figure 1b, where the ideal crossbar is assumed without any parasitic resistance.

Figure 1c shows a schematic of nonideal memristor crossbar, where parasitic crossbar resistance such as *R_S_*, *R_W_*, and *R_N_* are considered. *R_S_*, *R_W_*, and *R_N_* represent source resistance, line resistance, and neuron resistance, respectively. Here HRS and LRS are represented with white and gray boxes, respectively. Like Figure 1b, the nonideal memristor crossbar is used to store the synaptic weights of ANN in Figure 1a. The synaptic weights stored in the crossbar of Figure 1c are assumed binary (−1 and +1) or ternary (−1, 0, and +1), as mentioned in Figure 1b. In Figure 1c, *V*_*IN*,1_, *V*_*IN*,2_, and *V*_*IN*,*m*_ are input row voltages for Row #1, Row #2, and Row #m, respectively. Here ‘m’ means the number of rows in the crossbar. *V*_*S*,1_, *V*_*S*,2_, and *V*_*S*,*m*_ are source voltages on the rows, which are degraded due to *R_S_* and *R_W_*, for Row #1, Row #2, and Row #m, respectively. *I*_1_, *I*_2_, and *I_N_* are column currents of Column #1, Column #2, and Column #n, respectively. ‘n’ is the number of columns in the crossbar. Similarly, *V*_*N*,1_, *V*_*N*,2_, and V_N,n_ are neuron voltages on the columns affected due to *R_N_* and *R_W_*, for Column #1, Column #2, and Column #n, respectively. In the crossbar, *M*_11_ and *M*_12_ are transistors for controlling memristors of *R*_*M*,11_ and *R*_*M*,12_, respectively. *R*_*M*,11_ is a memristor cell connected with Row #1 and Column #1. *R*_*M*,12_ is a memristor cell connected with Row #1 and Column #2. The access-controlling transistors of *M*_11_ and *M*_12_ are turned on or off by the signals from the column control block, shown in Figure 1c.

The source, line, and neuron resistance in the nonideal crossbar in Figure 1c can affect the source voltages and the column currents. First, let us consider the nonideal effect due to source resistance. From Kirchhoff law, the source voltage, v.s. can be calculated by dividing the input voltage, *V_IN_*, between *R_S_* and the rest part of the row line including the line resistance and LRS cells along the row line. Usually, HRS cells affect the source voltage very little, because the conductance is negligibly small. If the number of LRS cells for a row becomes larger, the source voltage on the row line is affected more by the source resistance, *R_S_*, not by the LRS cells. This leads to the degradation of the source voltage. Similarly, we can consider the nonideal effect due to neuron resistance, *R_N_*. If the number of LRS cells for a column is increased larger and the parallel combination of LRS for the column becomes much smaller than *R_N_*, the column current begins to be dominated by *R_N_*, not by the parallel combination of LRS cells. If so, the column current is observed to be degraded compared to the column current of the ideal crossbar with *R_N_* = 0. A more detailed analysis of the nonideal effects due to *R_S_* and *R_N_* will be explained in the next section.

The degradation of source voltage and column current due to the nonideal effects such as source, line, and neuron resistance can affect neural network’s performance significantly. This is because the synaptic weights calculated from the backpropagation algorithm assume the memristor crossbar for the network is ideal not suffering the nonideal effects. To mitigate the performance gap between the ideal and nonideal crossbars, adaptive training methods have been proposed to consider the nonideal effects in the crossbar during the training phase [29,30]. For doing this, however, the training algorithm of the nonideal crossbar should be more complicated and the computational load becomes heavier to consider the voltage and current degradation due to the nonideal effects. The complicated training algorithm with heavy computational load is a big disadvantage, in terms of the training energy and time, particularly, for the on-device training applications such as edge devices, IoT sensors, etc.

Unlike the adaptive training methods, the compensation of the nonideal effects can be performed during the inference phase, not relying on the complicated training algorithm of the nonideal crossbar. The linear correction method was proposed to compensate for the nonideal effects during the inference time [10]. Though the computational burden becomes smaller compared to the adaptive training method, the linear correction proposed previously was performed by an external Trans-Impedance Amplifier (TIA) not being included in the memristor crossbar circuit [10]. The external TIA should be equipped with programmable gain and offset to calibrate the column current for compensating the nonideal effects [10]. The correction by the external TIA causes the calibration overhead because each TIA should be programmed with different gain and offset values for compensating the corresponding column’s nonideal behavior [10]. One more thing to note here is that the linear correction was proposed only for compensating the column current not the source voltage [10]. Unlike the previous linear correction by the external TIA, a new correction circuit proposed in this paper can be included in the memristor crossbar for compensating not only the column current but also the row voltage degradation due to the nonideal effects.

Particularly, implementing the nonideal-effect correction in the memristor crossbar circuit is very important for realizing the edge intelligence at IoT sensors, where the hardware and power overheads are critically important. For avoiding the hardware and power overheads due to the external hardware, the compensation of nonideal effects should be realized inside the memristor crossbar circuit not relying on the external hardware. For doing this, a new memristor-CMOS hybrid circuit for realizing the nonideal-effect correction is proposed in this paper. This proposed circuit can compensate for the nonideal effects in the inference phase, not in the training phase. Thus, the training algorithm in this paper can be as simple as the normal backpropagation of the ideal crossbar, not using the complicated adaptive training algorithm. In addition, the correction circuit is implemented in the memristor crossbar not using the external hardware. By doing so, the power and hardware overheads can be avoided in this paper. In the next section, a new neuron circuit with the nonideal-effect correction is explained in detail. In Section 3, the proposed correction neuron circuit is tested and discussed for MNIST (Modified National Institute of Standards and Technology database) and CIFAR-10 data sets [12,31]. In Section 4, we conclude this work finally.

**Figure 2:**
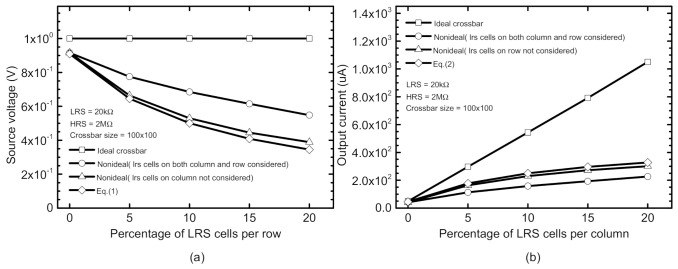
(**a**) Comparison of the source voltage of the ideal crossbar, the nonideal one (LRS cells on both column and row considered), the nonideal one (LRS cells on a column not considered), and the calculation with Equation (1). (**b**) Comparison of the output current of the ideal crossbar, the nonideal one (LRS cells on both column and row considered), the nonideal one (LRS cells on row not considered), and the calculation with Equation (2). Here, the ideal crossbar is assumed zero parasitic resistance. The nonideal crossbar is assumed with *R_S_* = 2 kΩ, *R_N_* = 2 kΩ, and *R_W_* = 1 Ω. The ideal and nonideal crossbars are simulated with a CADENCE SPECTRE version 6.1.6 circuit simulator.

Figure 2a shows the source voltage degradation due to the nonideal effects such as *R_S_*, *R_N_*, and *R_W_*, as mentioned just earlier. In the ideal crossbar, v.s. seems constant despite increasing the percentage of LRS cells among all the memristor cells per row. On the contrary, the source voltage in the nonideal crossbar is degraded with increasing the percentage of LRS per row, as shown in Figure 2a. Assuming the source voltage is affected little by *R_W_* and *R_N_*, the source voltage of Row #i, *V*_*S*,*i*_ can be simply approximated with the following equation.

## 2. Method

Here **V*_*IN*,*i*_* is the input voltage of Row #i and assumed as large as 1 V, as shown in Figure 2a. In Equation (1), *G_S_* means the inverse of source resistance, *R_S_*. *G_LRS_* and *G_HRS_* are the conductance of LRS and HRS cells, respectively. ‘*l_i_*’ means the number of LRS cells of Row #i. ‘n’ means the number of columns in the crossbar. Thus, ‘*n*−*l_i_*’ represents the number of HRS cells of Row #i in Equation (1). In Figure 2a, the X-axis is the percentage of LRS cells among all the memristor cells of Row #i. Here the percentage of LRS of Row #i is calculated with ‘*l_i_*/*n*(%)’. As expected from Equation (1), *V*_*S*,*i*_ is degraded with increasing the number of LRS cells for Row #i. This is because the voltage drop on *R_S_* becomes larger, as the number of LRS cells of the row is increased. In Figure 2a, we compared the source voltage of the ideal crossbar, the nonideal one (LRS cells on both column and row considered), the nonideal one (LRS cells on a column not considered), and the calculation with Equation (1). Here, the nonideal crossbar is assumed with *R_S_* = 2 kΩ, *R_N_* = 2 kΩ, and *R_W_* = 1 Ω. The ideal and nonideal crossbars are simulated with a CADENCE SPECTRE version 6.1.6 circuit simulator. In Figure 2a, Equation (1) is calculated with MATLAB. The gap between the Equation (1) and the nonideal crossbar (LRS cells on a column not considered) is very little. This is because *R_W_* = 1Ω can affect the source voltage very little. Here the end-to-end line resistance is as small as 100 Ω, for 100 × 100 crossbar. Compared to the end-to-end line resistance of 100 Ω, *R_S_* is as large as 2 kΩ in Figure 2a. The nonideal crossbar (LRS cells on both column and row considered) shows a larger source voltage than Equation (1). This is because more LRS cells on the column can boost up the source voltage higher than Equation (1).

(1) $$V_{S,i}\approx \frac{G_{S}\cdot V_{IN,i}}{G_{S}+\frac{l_{i}}{R_{LRS}+R_{N}}+\frac{n-l_{i}}{R_{HRS}+R_{N}}}$$

The column current can be changed due to *R_S_*, *R_N_*, and *R_W_* like the source voltage, too. Figure 2b shows the column current degradation due to the nonideal effects. For the ideal crossbar with zero parasitic resistance, the column current seems proportional to the number of LRS cells per column. However, as indicated in Figure 2b, the column current seems saturated due to *R_N_*, in the nonideal crossbar. Similarly, with Equation (1), assuming the column current is affected little by *R_S_* and *R_W_*, the column current can be expressed with

(2) $$I_{j}\approx \frac{G_{N}\cdot \sum_{i=1}^{m}\left(G_{M,ij}\cdot V_{S,i}\right)}{G_{N}+\frac{k_{j}}{R_{LRS}+R_{S}}+\frac{m-k_{j}}{R_{HRS}+R_{S}}}$$

*I_j_* is the column current for Column #j. *G_N_* is the inverse of *R_N_*. *G*_*M*,*ij*_ is memristor’s conductance for Row #i and Column #j. *V*_*S*,*i*_ is the source voltage of Row #i, as mentioned in Equation (1). The term of $\sum_{i=1}^{m}\left(G_{M,ij}\cdot V_{S,i}\right)$ calculates the summation of the conductance-voltage multiplications from Row #1 to Row #n, for Column #j. ‘m’ is the number of rows in the crossbar. In the denominator term, ‘*k_j_*’ is the number of LRS cells for Column #j. Thus, ‘m-*k_j_*’ means the number of HRS cells for Column #j.

The percentage of LRS cells for Column #j is calculated with ‘*k_j_*/m(%)’. In the ideal crossbar, it is expected that the column current, Ij is proportional to the number of LRS cells for its own column. However, in the nonideal crossbar with parasitic neuron resistance *R_N_*, the column current begins to be saturated, when the number of LRS cells is increased. From Equation (2), it is obvious that the saturation comes from the term *k_j_* · *G_LRS_* in the denominator term. In Figure 2b, we compared the source voltage of the ideal crossbar, the nonideal one (LRS cells on both column and row considered), the nonideal one (LRS cells on a column not considered), and the calculation with Equation (2). Here, the nonideal crossbar is assumed with *R_S_* = 2 kΩ, *R_N_* = 2 kΩ, and *R_W_* = 1 Ω. The ideal and nonideal crossbars are simulated with a CADENCE SPECTRE version 6.1.6 circuit simulator. In Figure 2b, Equation (2) is calculated with MATLAB. Like Figure 2a, the gap between the Equation (2) and the nonideal crossbar (LRS cells on row not considered) seems very small. This is because *R_W_* = 1 Ω can affect the column current very little. Here the end-to-end line resistance is as small as 100 Ω, for 100 × 100 crossbar. Compared to the end-to-end line resistance of 100 Ω, *R_N_* is as large as 2 kΩ in Figure 2b. The nonideal crossbar (LRS cells on both column and row considered) shows a lower column current than Equation (2). This is because more LRS cells on a row can suppress the column current more severely than Equation (2).

Here the memristor array size simulated in Figure 2a,b is assumed 100 rows and 100 columns. Here LRS and HRS are assumed 20 kΩ and 2 MΩ, respectively.

**Figure 3:**
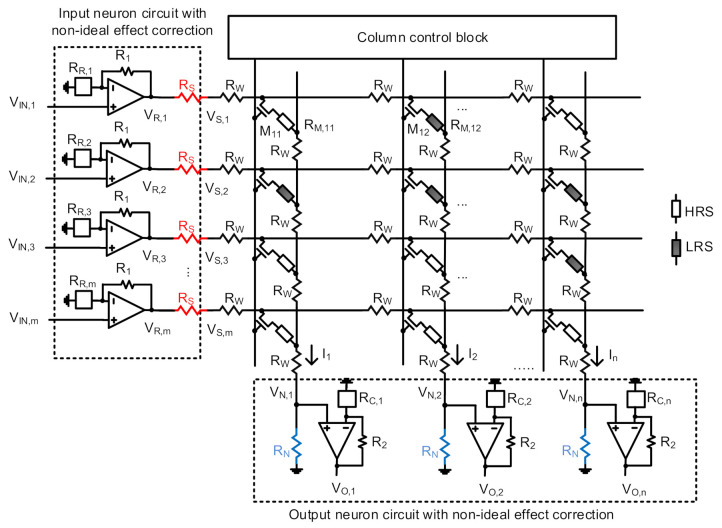
Schematic of the proposed memristor crossbar circuit with correction of nonideal effects. Here the nonideal-effect correction circuit in the left is for compensating the loss of source voltage. The correction circuit added to the bottom is for reducing the loss of output voltage.

To compensate for the voltage and current degradation due to the nonideal effects in the nonideal crossbar, a new memristor-CMOS hybrid circuit for realizing the nonideal-effect correction neuron is proposed, as indicated in Figure 3. The proposed circuit in Figure 3 can correct both the source voltage and column current degradation from the nonideal effects, in the inference phase, not in the training phase. Thus, the training procedure in this paper can be as simple as the normal backpropagation of the ideal neural networks, not using the complicated adaptive training algorithm [30,31]. In addition, the correction can be implemented in the memristor crossbar circuit in Figure 3 without using the complicated digital control block such as the external correction method [10]. By doing so, the power and hardware overhead due to the external digital controller can be avoided in the proposed neuron circuit with nonideal-effect correction.

Figure 3 shows a schematic of the memristor crossbar circuit, where the proposed correction circuit is added for compensating the nonideal effects due to source and neuron resistance. Here the memristor crossbar is used to store the synaptic weights of neural networks. In Figure 3, memristor cells with HRS and LRS are represented with open and solid styles, respectively. *R_W_*, *R_S_*, and *R_N_* mean parasitic line, source, and neuron resistance of the nonideal crossbar, respectively. In Figure 3, *M*_11_ and *M*_12_ are transistors for accessing the memristors of *R*_*M*,11_ and *R*_*M*,12_, respectively. *R*_*M*,11_ is an HRS cell connected with Row #1 and Column #1. *R*_*M*,12_ is the LRS cell connected with Row #1 and Column #2. The access transistors of *M*_11_ and *M*_12_ are turned on or off by the signals from the column control block, shown in Figure 3.

Looking at the row lines in Figure 3, *V*_*IN*,1_, *V*_*IN*,2_, and *V*_*IN*,*m*_ are external input voltages applied to Row #1, Row #2, and Row #m, respectively. The input voltages enter the correction circuit, where the input voltages are converted to the correction voltages. The correction circuit for Row #1 is made of the non-inverting amplifier with *R*_1_ and *R*_*R*,1_. Similarly, the correction circuit for Row #2 has the noninverting amplifier with *R*_1_ and *R*_*R*,2_. Here it should be noted that *R*_1_ has the same resistance value for all the rows in the crossbar, while *R*_*R*,*i*_ made of a memristor can be programmed with a different value for each row. *V*_*R*,1_, *V*_*R*,2_, and V_R,m_ represent the correction voltages for compensating the nonideal effect due to source resistance. *V*_*R*,*i*_ for Row #i, can be calculated with $\left(1+\frac{G_{R,i}}{G_{1}}\right)\cdot V_{IN,i}$. *G*_*R*,*i*_, and *G*_1_ are the inverse of *R*_*R*,*i*_ and *R*_1_, respectively. After passing through source resistance of *R_S_*, *V*_*S*,1_, *V*_*S*,2_, and *V*_*S*,*m*_ represent the source voltages for Row #1, Row #2, and Row #m, respectively. Using Equation (1), the source voltage of Row #i in Figure 3 can be approximated with

(3) $$V_{S,i}\approx \frac{G_{S}\cdot \left(1+\frac{G_{R,i}}{G_{1}}\right)\cdot V_{IN,i}}{G_{S}+\frac{l_{i}}{R_{LRS}+R_{N}}+\frac{n-l_{i}}{R_{HRS}+R_{N}}}=\frac{\left(1+\frac{G_{R,i}}{G_{1}}\right)\cdot V_{IN,i}}{\left(1+l_{i}\cdot \frac{1}{G_{S}}\cdot \left(\frac{1}{R_{LRS}+R_{N}}-\frac{1}{R_{HRS}+R_{N}}\right)+n\cdot \frac{1}{G_{S}\left(R_{HRS}+R_{N}\right)}\right)}$$

As mentioned in Equation (1), ‘*l_i_*’ and ‘*n*−*l_i_*’ are the numbers of LRS and HRS cells for Row #i, respectively. ‘n’ means the number of columns in the crossbar. *G_S_* is the inverse of *R_S_*. The nonideal effect due to *R_S_* is caused by $l_{i}\cdot \frac{1}{G_{S}}\cdot \left(\frac{1}{R_{LRS}+R_{N}}-\frac{1}{R_{HRS}+R_{N}}\right)$ in the denominator term of Equation (3). From this equation, the source voltage, *V*_*S*,*i*_ is expected to be lowered, as the number of LRS cells for Row #i becomes large. By adjusting the resistance *R*_*R*,*i*_ for Row #i, in the noninverting op amp, we can make the condition of $\frac{G_{R,i}}{G_{1}}\approx l_{i}\cdot \frac{1}{G_{S}}\cdot \left(\frac{1}{R_{LRS}+R_{N}}-\frac{1}{R_{HRS}+R_{N}}\right)$. By doing so, the source voltage loss due to source resistance can be compensated in Equation (3). Here it should be noted that *R*_*R*,*i*_ of Row #i in Figure 3 can be made of a memristor.

Looking at the column lines in Figure 3, *I*_1_, *I*_2_, and *I_N_* are the column currents for Column #1, Column #2, and Column #n, respectively. *V*_*N*,1_, *V*_*N*,2_, and V_N,n_ are the neuron voltages for Column #1, Column #2, and Column #n, respectively, which are generated from the column currents. *V*_*O*,1_, *V*_*O*,2_, and *V*_*O*,*n*_ are the corrected column voltages for taking into account the parasitic neuron resistance. The correction circuit for Column #1 is composed of the non-inverting amplifier with *R*_*C*,1_ and *R*_2_. The correction circuit for the next Column #2 has *R*_*C*,2_ and *R*_2_. Here it should be noted that *R*_2_ has the same resistance for all the columns in the crossbar, while *R*_*C*,*j*_ made of a memristor can be programmed with a different value for each column. The corrected output voltage of *V*_*O*,*j*_ for Column #j can be calculated with $\left(1+\frac{G_{C,j}}{G_{2}}\right)\cdot V_{N,j}$. *G*_*C*,*j*_ and G_2_ are the inverse of *R*_*C*,*j*_ and *R*_2_, respectively. Using *V*_*N*,*j*_ from the Equation (2), the output voltage of the correction circuit for Column #j is expressed roughly with

(4) $$\begin{array}{ll} V_{O,j}\approx V_{N,j}\cdot \left(1+\frac{G_{C,j}}{G_{2}}\right) & \approx \frac{\sum_{i=1}^{m}\left(G_{M,ij}\cdot V_{S,i}\right)\cdot \left(1+\frac{G_{C,j}}{G_{2}}\right)}{G_{N}+\frac{k_{j}}{R_{LRS}+R_{S}}+\frac{m-k_{j}}{R_{HRS}+R_{S}}}\\ & =\frac{\sum_{i=1}^{m}\left(G_{M,ij}\cdot V_{S,i}\right)\cdot \left(1+\frac{G_{C,j}}{G_{2}}\right)}{G_{N}\left(1+k_{j}\cdot \frac{1}{G_{N}}\cdot \left(\frac{1}{R_{LRS}+R_{S}}-\frac{1}{R_{HRS}+R_{S}}\right)+m\cdot \frac{1}{G_{N}\left(R_{HRS}+R_{S}\right)}\right)} \end{array}$$

As mentioned in Equation (2), ‘*k_j_*’ and ‘m-*k_j_*’ are the numbers of LRS and HRS cells for Column #j, respectively. ‘m’ means the number of rows in the crossbar. *G_N_* is the inverse of *R_N_*. Similarly, with Equation (3), the output voltage degradation due to *R_N_* is caused from $k_{j}\cdot \frac{1}{G_{N}}\cdot \left(\frac{1}{R_{LRS}+R_{S}}-\frac{1}{R_{HRS}+R_{S}}\right)$ in the denominator term of Equation (4). As a column has more LRS cells, the column current becomes degraded more significantly. If *G*_*C*,*j*_ is adjusted according to the number of LRS cells for Column #j, the degradation due to $k_{j}\cdot \frac{1}{G_{N}}\cdot \left(\frac{1}{R_{LRS}+R_{S}}-\frac{1}{R_{HRS}+R_{S}}\right)$ can be reduced. Here *G*_*C*,*j*_ implemented with a memristor can be programmed with different values to adjust its conductance for compensating the column current loss due to neuron resistance.

**Figure 4:**
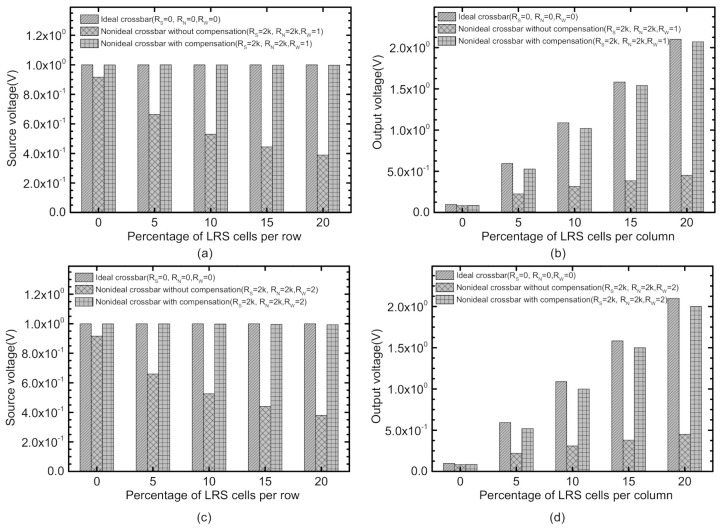
(**a**) Comparison of the source voltages of the nonideal crossbars between without and with the correction circuit for *R_S_* = 2 kΩ and *R_W_* = 1 Ω per cell. (**b**) Comparison of the output voltages of the nonideal crossbars between without and with the correction circuit for *R_N_* = 2 kΩ and *R_W_* = 1 Ω per cell. (**c**) Comparison of the source voltages of the nonideal crossbars between without and with the correction circuit for *R_S_* = 2 kΩ and *R_W_* = 2 Ω per cell. (**d**) Comparison of the output voltages of the nonideal crossbars between without and with the correction circuit for *R_N_* = 2 kΩ and *R_W_* = 2 Ω per cell.

Figure 4a–d compares the ideal crossbar, the nonideal without compensation, and the nonideal with compensation. Among these four figures, Figure 4a,b indicate the source and output voltages, respectively, for *R_W_* = 1Ω. Similarly, Figure 4c,d show the simulated source and output voltages for *R_W_* = 2Ω. The source voltage compensation is achieved by the correction circuit shown on the left in Figure 3. The correction of the column’s output voltage is performed by the circuit shown at the bottom in Figure 3.

Figure 4a indicates the source voltage can be compensated by using the correction circuit in Figure 3. Here, the nonideal crossbar without the correction shows the source voltage becomes degraded as small as 38.9% compared to the ideal crossbar when the percentage of LRS cells per row is 20%. However, the correction circuit added to the nonideal crossbar can improve the source voltage from 38.9% to 99.7% of the ideal crossbar, as shown in Figure 4a.

Figure 4b compares the output voltages of the three crossbar circuits, which are the ideal crossbar, the nonideal one without the correction circuit, and the nonideal one with the correction circuit, respectively. The correction circuit used in Figure 4b is shown at the bottom in Figure 3. The output voltage of the ideal crossbar is proportional to the number of LRS cells per column. However, the nonideal crossbar without compensation indicates the output voltage begins to be saturated when the number of LRS cells per column is increased. The correction circuit can restore the output voltage from 21.5% to 98.5% of the ideal crossbar when the percentage of LRS cells per row is 20%.

In Figure 4c,d, the ideal crossbar, the nonideal without compensation, and the nonideal with compensation, are simulated for *R_W_* = 2 Ω. As expected, *R_W_* = 2 Ω degrades the source and output voltages more severely than *R_W_* = 1 Ω. In Figure 4c, the nonideal without compensation has the source voltage as small as only 38%, compared to the ideal crossbar. The correction circuit can recover the output voltage from 38% to 99.3% of the ideal crossbar, as indicated in Figure 4c. Similarly, Figure 4d shows the output voltage of the uncompensated crossbar is as small as 21.4% of the ideal crossbar. If the correction circuit is used in Figure 4d, the output voltage can reach as large as 95.2% of the ideal crossbar, when the percentage of LRS cells per column is assumed 20%.

**Figure 5:**
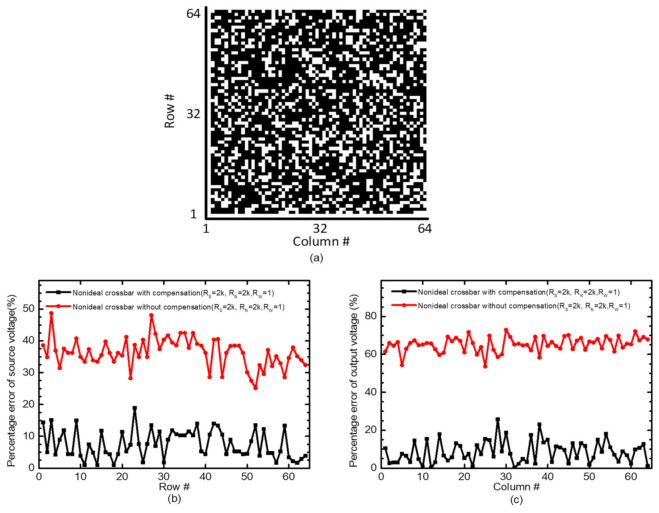
(**a**) Memristor crossbar with 64 rows and 64 columns. Here the white and black pixels represent LRS and HRS, respectively. (**b**) The percentage error of source voltage from Row #1 to Row #64. Here *R_S_* = 2 kΩ, *R_N_* = 2 kΩ, and *R_W_* = 1 Ω. The percentage error between the ideal and nonideal without the correction circuit is shown in the red line. The percentage error with the correction is shown in the black line. (**c**) The percentage error of output voltage from Column #1 to Column #64.

In Figure 5, the percentage errors between the ideal and nonideal crossbars are simulated using the CADENCE SPECTRE version 6.1.6 circuit simulator. The simulated memristor crossbar is assumed to be 64 rows and 64 columns. Here the white and black pixels represent LRS and HRS, respectively, in Figure 5a.

Figure 5b indicates the percentage error of source voltage from Row #1 to Row #64. Here *R_S_* = 2 kΩ, *R_N_* = 2 kΩ, and *R_W_* = 1 Ω. The percentage error between the ideal and nonideal without the correction circuit is shown in the red line. The percentage error with the correction is shown in the black line, in Figure 5b. The average percentage error of the uncompensated crossbar is as large as 36.7%. If the correction circuit is used, the percentage error in the source voltage can be reduced from 36.7% to 7.5%. Figure 5c shows the percentage error of output voltage from Column #1 to Column #64. The average percentage error of the uncompensated crossbar is as large as 65.5%. The correction circuit can improve the percentage error from 65.5% to 8.6%. Almost the percentage error can be reduced to ~1/7, if the correction circuit is used.

## 3. Results

**Figure 6:**
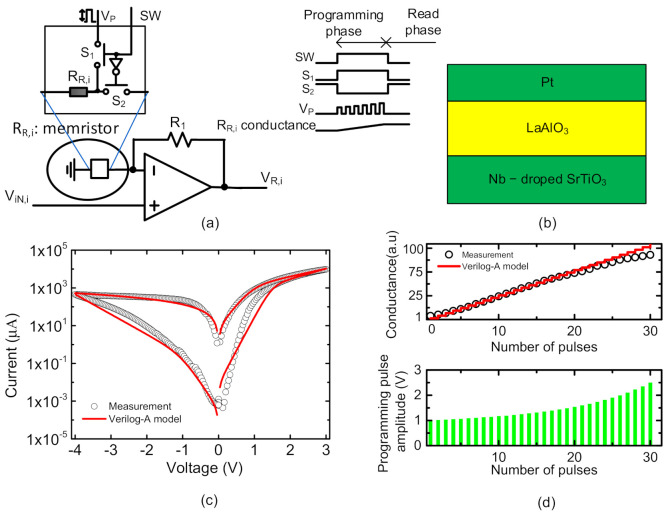
(**a**) Schematic of the correction circuit for source voltage of Row #i. Here *R*_*R*,*i*_ is implemented with a memristor device. (**b**) Cross-sectional view of the memristor measured in this paper. (**c**) Comparison of the measurement and simulated Verilog-A model of the memristor device in Figure 6b. (**d**) Memristor programming for adjusting its conductance with increasing the number of programming pulses.

Figure 6a shows a simple schematic of the proposed correction circuit for compensating the voltage loss due to the nonideal effects, which was already shown in the left and bottom in Figure 3. *R*_1_ represents feedback resistance of noninverting op amp. It should be noted *R*_1_ has the same resistance for all the rows in the nonideal crossbar. In Figure 6a, *V*_*IN*,*i*_ means the input voltage for Row #i. *V*_*R*,*j*_ is the compensated voltage by the correction circuit. As explained earlier, the *V*_*R*,*i*_ can be obtained with $V_{R,i}=\left(1+G_{R,i}/G_{1}\right)\cdot V_{IN,i}$, where *R*_*R*,*i*_ can be made of a memristor cell for adjusting its conductance according to the number of LRS cells for Row #i.

The white box in Figure 6a shows the memristor, *R*_*R*,*i*_ with the programming circuit. *R*_*R*,*i*_ is put between the minus terminal and ground node. *R*_*R*,*i*_ should be programmed with different conductance values for each row according to the number of LRS cells for the corresponding row. SW is a switch control signal for programming *R*_*R*,*i*_. *S*_1_ and *S*_2_ are the switches for programming and reading operations of the memristor, respectively. *V_P_* is a memristor programming pulse train voltage, which can be modulated for linear and precise programming of memristor’s conductance, as explained in Figure 6d. The simple waveforms of the memristor programming circuit are also shown in Figure 6a, where the memristor’s conductance is changed according to the programming pulse train, *V_P_*, during the programming phase. In the read phase, the memristor’s conductance is used to compensate for the nonideal effect.

Figure 6b shows the cross-sectional view of the memristor device used in this paper. The memristor in Figure 6b has a film structure composed of Pt/LaAlO3/Nb-doped SrTiO stacked layers [18,20]. Figure 6c shows the measurement and the Verilog-A model for the memristor device in Figure 6b. The measured data are represented with open circles. The calculated current-voltage relationship from the Verilog-A model is shown in the red line. For the entire operation region, the Verilog-A model of the memristor is in good agreement with the measured data. In Figure 6c, Keithley-4200 (Semiconductor Characterization System) was used for the measurement of the current-voltage relationship of the memristor device. The model equations for the memristor device in Figure 6b are programmed with Verilog-A language and simulated by CADENCE SPECTRE version 6.1.6 circuit simulator in Figure 6c. The model equations used in Figure 6c were explained in detail in the previous publication [18]. For simulating the hybrid circuit of memristors and CMOS devices, we need to have CMOS parameters, in this paper. The CMOS model parameters used here were obtained from the 0.18-um CMOS process of CMOS logic Foundry Company. Figure 6d shows the programmed conductance of the memristor device according to the number of programming pulses. Here, the program-verify method with the fine Pulse Amplitude Modulation (PAM) is used for adjusting the conductance of the memristor precisely, as shown in Figure 6d. As the number of fine-modulated programming pulses is increased, the conductance of the memristor seems to change in proportion to the number of programming pulses [18]. This linear relationship between the programmed conductance and the number of programming pulses is very helpful in adjusting *R*_*R*,*i*_ accurately in Figure 6a.

One more concern of programming *R*_*R*,*i*_ may be endurance. Fortunately, the memristor in the correction circuit is programmed once according to the number of LRS cells of the corresponding row during the training phase. In the inference operation, the memristor *R*_*R*,*i*_ in the correction circuit is only read not be programmed. The endurance cycles have been experimentally measured within 10^5^–10^7^, which can be enough not only for the inference phase but also for the training phase [10,32].

**Figure 7:**
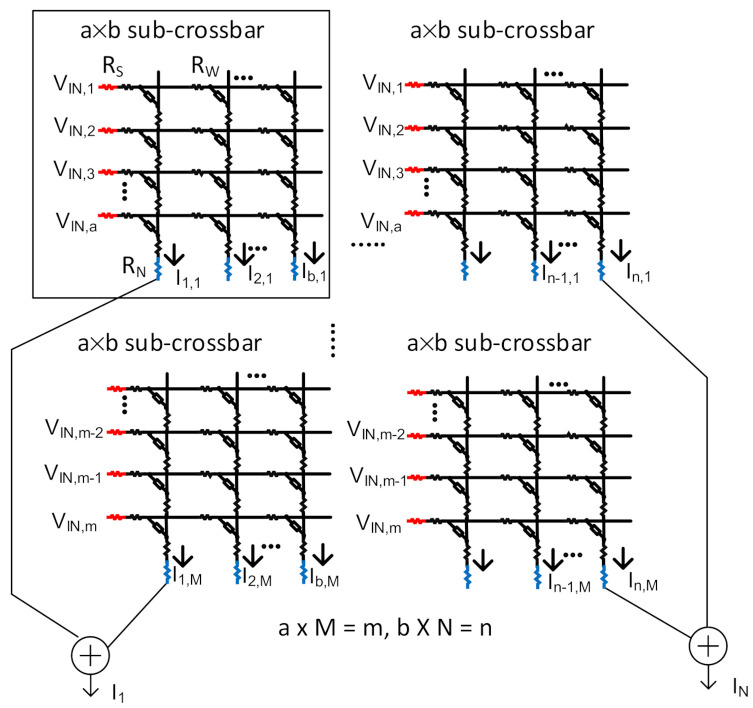
Tile architecture of multiple sub-crossbars for implementing a memristor crossbar with ‘m × n’ cells. Here each sub-crossbar size is ‘a × b’. The numbers of sub-crossbars for rows and columns are ‘M’ and ‘N’, respectively. ‘M’ and ‘N’ can be calculated from ‘m = M × a’ and ‘n = N × b’, respectively. Here RS, RW, and RN are the source, line, and neuron resistance, respectively, contributing to the nonideal effects in the memristor crossbar.

Figure 7 shows a tiled architecture of memristor crossbar composed of sub-crossbars for implementing a large network [30,32,33] For designing a neural network with a large number of neurons and synapses, it is better to put together sub-crossbars, not building a single big crossbar.

The tile architecture in Figure 7 is very useful in limiting the nonideal effects under a certain level. Usually, the size of sub-crossbar is within 128 × 128 array [30,32,33]. In this paper, we used the sub-crossbar with 100 × 100 memristor cells. In Figure 7, a sub-crossbar has ‘a × b’ memristor cells. ‘a’ and ‘b’ mean the numbers of rows and columns for sub-crossbar, respectively. If the total number of rows and columns needed for implementing a neural network is ‘m × n’, ‘m’ and ‘n’ can be calculated simply with ‘m = M × a’ and ‘n = N × b’, respectively [30]. Here, ‘M’ and ‘N’ are the numbers of sub-crossbars for rows and columns, respectively. For example, if we try to implement the MNIST neural network with 784 input neurons and 200 hidden neurons, ‘M’ and ‘N’ should be 8 and 2, respectively.

Considering practical parameters of memristor crossbars, we used LRS = 20 kΩ and HRS = 2 MΩ, for the circuit simulation, in this paper. These LRS and HRS values are obtained from the experimental parameters of real fabricated crossbars [30]. The line resistance used in the circuit simulation is 1 Ω per cell obtained from TSMC RRAM data [33]. The sub-crossbar size used in the MNIST and CIFAR-10 simulation is 100 × 100 [30,32,33].

To estimate the neural network’s performance realized with the nonideal-effect correction, the memristor crossbar in Figure 3 is tested here for the MNIST dataset [12]. The MNIST data set is composed of 60,000 training images and 10,000 testing ones. Each image has 28 × 28 = 784 pixels. The classified items are hand-written digits from ‘0′ to ‘9′. Thus, each digit has 6000 training and 1000 testing vectors. The neural network simulated here for testing the MNIST dataset has 784 input and 200 hidden neurons. The number of output neurons is 10. The fully connected neural network (784-200-10 neurons) for testing MNIST vectors is implemented with the tiled architecture of multiple sub-crossbars, as shown in Figure 7.

**Figure 8:**
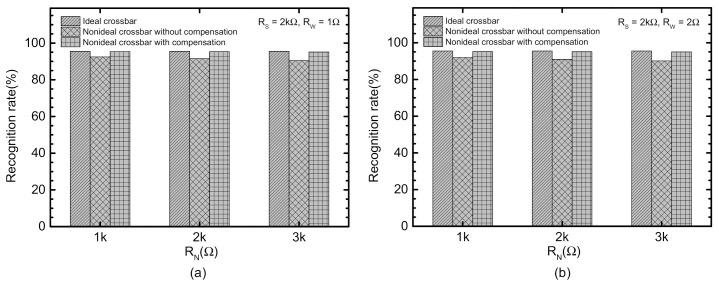
(**a**) Comparison of the recognition rate of the nonideal crossbars between without and with the correction circuit for MNIST data set with *R_W_* = 1 Ω, (**b**) Comparison of the recognition rate of the nonideal crossbars between without and with the correction circuit for MNIST data set with *R_W_* = 2 Ω.

Figure 8a compares the recognition rate of the ideal crossbar, the nonideal without the correction, and the nonideal with the correction circuit. Here the source and line resistance are assumed 2 kΩ and 1 Ω, respectively. The neuron resistance is increased from 1 kΩ to 3 kΩ. In Figure 8a, the first, second, and third bars are for the ideal, the nonideal without correction, and the nonideal with correction, respectively. When the neuron resistance is 1 kΩ, the uncompensated crossbar and compensated one indicates the recognition rate, 92.5% and 95.4%, respectively, compared to the rate of the ideal crossbar, 95.5%. Here, it should be noted that the correction circuit could improve the rate of the compensated crossbar by 2.9% compared to the uncompensated. If the neuron resistance is as large as 3 kΩ, the rate of the crossbars without and with the correction circuit are 90.4% and 95.1%, respectively. The gap in recognition rate between the uncompensated and compensated crossbars is increased from 2.9% to 4.7%, as shown in Figure 8a. One thing to note here is that the recognition rate of 95.5% in Figure 8a seems lower than the state-of-the-art rate as high as 99%. This is mainly due to the ternary synaptic weights used in the memristor crossbar instead of the floating-point numbers. When the memristor crossbar is tested with the integer synaptic weights, the rate could be as high as 98% for MNIST vectors.

Similarly, Figure 8b compares the nonideal crossbars without and with the correction circuit, for *R_W_* = 2 Ω. Here RW in Figure 8b is 2× larger than *R_W_* in Figure 8a. The recognition rate without the correction is as small as 90.1%. However, the correction circuit can increase the rate as large as 95%. The gap between without and with the correction circuit is 4.9% in Figure 8b.

Next, the proposed correction circuit is tested for the CIFAR-10 (Canadian Institute For Advanced Research) data set. Like the MNIST dataset, CIFAR-10 has 50,000 training images and 10,000 testing ones for classifying 10 image objects. Each image is composed of 32 × 32 RGB pixels. For each image object, there are 5000 training and 1000 testing vectors. The ten image objects are airplane, automobile, bird, cat, deer, dog, frog, horse, ship, and truck. Here the ResNet CNN architecture is used for testing the CIFAR-10 dataset [34]. Here only the fully-connected layers in the ResNet are implemented with the tile architecture of sub-crossbars in Figure 7. Here, in the fully connected layer in ResNet, the number of hidden neurons is 200. The output neurons for the neural network should be 10 for classifying the 10 image objects such as airplanes, automobiles, and so on in the CIFAR-10 dataset.

**Figure 9:**
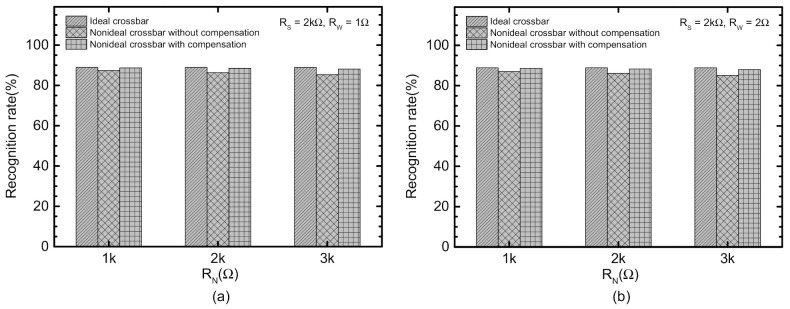
(**a**) Comparison of the recognition rate of the nonideal crossbars between without and with the correction circuit for CIFAR-10 data set with *R_W_* = 1 Ω (**b**) Comparison of the recognition rate of the nonideal crossbars between without and with the correction circuit for CIFAR-10 data set with *R_W_* = 2 Ω.

Figure 9a compares the recognition rate of the ideal crossbar, the nonideal without the correction, and the nonideal with correction circuit, for the CIFAR-10 dataset. In Figure 9a, the first, second, and third bars are for the ideal, the nonideal without correction, and the nonideal with correction, respectively. When the neuron resistance is as small as 1 kΩ, the uncompensated crossbar and compensated one indicates the recognition rate, 87.4% and 88.7%, respectively, compared to the rate of the ideal crossbar, 88.9%. The gap between the uncompensated and the compensated is as small as 1.3% for the neuron resistance = 1 kΩ. For *R_N_* = 3 kΩ, the correction circuit seems to improve the recognition rate from 85.3% to 88.1%, compared to the nonideal without compensation. The gap between the uncompensated and compensated becomes as large as 2.8%, for *R_N_* = 3 kΩ.

Figure 9b indicates the recognition rate of the nonideal crossbars without and with the correction circuit, for *R_W_* = 2Ω. For *R_N_* = 3 kΩ, the recognition rate without the correction is as small as 85.1%. However, the correction circuit can increase the rate as large as 88%. The gap between without and with the correction circuit is as large as 2.9% in Figure 9b, for *R_N_* = 3 kΩ.

One more thing to discuss here is the power overhead due to the correction circuit in Figure 3. Using the circuit simulator of CADENCE SPECTRE version 6.1.6, the power consumption of the correction circuit is estimated at roughly ~0.76 uA for each noninverting amplifier. The power overhead ratio due to the correction circuit can be approximated with the following equation.

(5) $$R_{overhead}\approx \frac{\left(m+n\right)\cdot V_{DD}\cdot I_{correction,avg}}{m\cdot n\cdot V_{DD}\cdot \left(I_{LRS,avg}\cdot \alpha +I_{HRS,avg}\cdot \left(1-\alpha \right)\right)}$$

Here *R_overhead_* means the overhead ratio of the power due to the correction circuit with respect to the power consumption of the memristor crossbar. ‘m’ and ‘n’ are the numbers of rows and columns, respectively, in the crossbar. *I_correction,avg_* means the average current consumption for each noninverting op amp used in the correction circuit. VDD is the power supply voltage. *I_LRS,avg_* and *I_HRS,avg_* are the average currents for LRS cells and HRS cells in the crossbar, respectively. ‘α’ means the activity ratio of LRS cells during the inference phase. ‘1 − α’ is the activity ratio of HRS cells during the inference time. As expected in Equation (5), the overhead ratio depends on the crossbar size with ‘m’ and ‘n’. Assuming LRS = 20 kΩ and HRS = 2 MΩ, the overhead ratio is estimated at less than 1%. Here the sub-crossbar size is assumed 100 ×100. The activity ratio of LRS cells is assumed at 30% on average, which is obtained from the synaptic weights calculated from MATLAB simulation. If LRS becomes higher, the overhead ratio in Equation (5) becomes higher, too.

In addition, it should be discussed the power overhead due to the mixed-mode interface circuit such as an analog-to-digital (ADC) converter [32,35]. As mentioned, the memristor crossbar can perform VMM operation based on the analog current-voltage relationship of memristors. For performing the VMM operation in memristor crossbars, the mixed-mode interface circuit should be added to the memristor crossbar. The ADC is used for converting the VMM result to the digital data for delivering them to the other networks. For doing this, the ADC should consume an amount of computing power. Comparing the power consumption between the mixed-mode interface circuit and the memristor crossbar, it has been reported that the mixed-mode interface circuit including ADC consumes 9× larger power than the memristor crossbar [32]. Though the ADC consumes most of the computing power, the overall computational energy efficiency of the memristor crossbar-based neural networks can easily outperform the conventional digital-based neural networks relying on the digital VMM operation [35]. Thus, despite the power overhead of the mixed-mode interface circuit, the memristor crossbar-based neural networks can be very suitable particularly for the edge intelligence hardware such as IoT sensors, where energy-efficient computing is very critical [13,35].

Finally, it should be mentioned that we focused on solving the nonideal-effect problems related to the parasitic resistance such as source resistance, neuron resistance, and line resistance, in this paper. Of course, other nonideal effects are not considered in this paper, such as yield-limited memristor defects, nonlinear current-voltage behaviors, etc. [36,37]. Actually, for considering the yield-limited memristor defects, a defect-aware in-situ crossbar training can be used with the parasitic-resistance correction circuit proposed in this paper [37]. By putting the defect-aware training and parasitic-resistance correction circuit together, the nonideal-effect correction circuit can compensate the voltage and current loss due to the parasitic resistance and the defect-aware training can compensate the performance loss due to memristor defects such as stuck-at-0 and stuck-at-1 simultaneously.

For the nonideal-effect correction circuit shown in Figure 3, the added OP amp may have the offset voltage problem and the added resistors such as RR and RC may suffer process variation. However, the nonideal effects caused by the OP amp’s offset voltage and added RR and RC variations can be thought to cause smaller degradation of neural network’s performance than the gain of the recognition rate with the correction circuit. From the MNIST simulation, the variations of OP amp’s offset voltage, RR, and RC can degrade the MNIST recognition rate by about ~0.6%. This rate loss of ~0.6% is much smaller than ~4.7% that is the gain of the recognition rate due to the correction circuit.

## 4. Conclusions

The nonideal effects such as parasitic source, line, and neuron resistance can affect the input voltage and the output current of the memristor crossbar significantly. The nonideal effects cause the degradation of the neural network’s performance realized with the nonideal memristor crossbar. To avoid performance degradation due to the nonideal effects, the adaptive training methods were proposed previously [29,30]. However, the complicated training algorithm can add a heavy computational burden to the neural network hardware. Especially, this hardware and algorithmic burden can be more serious for edge intelligence applications such as IoT sensors.

In this paper, the memristor-CMOS hybrid neuron circuit was proposed for compensating the voltage loss due to the nonideal effects during the inference phase, not the training phase. Unlike the previous linear correction method performed by the external hardware [10], the proposed memristor-CMOS hybrid neuron circuit can be included in the memristor crossbar to minimize the power and hardware overheads caused by the nonideal-effect correction.

The proposed correction circuit was verified to be able to restore both the source voltage and the output voltage degradation due to the nonideal effects. For the source voltage, the average percentage error of the uncompensated crossbar is as large as 36.7%. If the correction circuit is used, the percentage error in the source voltage can be reduced from 36.7% to 7.5%. For the output voltage, the average percentage error of the uncompensated crossbar is as large as 65.2%. The correction circuit can improve the percentage error in the output voltage from 65.2% to 8.6%. Almost the percentage error can be reduced to ~1/7 if the correction circuit is used.

The nonideal memristor crossbar with the correction circuit was tested for MNIST and CIFAR-10 data sets in this paper. For MNIST, the uncompensated and compensated crossbars indicated the recognition rate of 90.5% and 95.1%, respectively, compared to 95.5% of the ideal crossbar. For CIFAR-10, the nonideal crossbars without and with the nonideal effect correction showed the rate of 85.4% and 88.3%, respectively, compared to the ideal crossbar not suffering the nonideal effects achieving the rate as large as 88.9%.
